# The right ventricular fibroblast secretome drives cardiomyocyte dedifferentiation

**DOI:** 10.1371/journal.pone.0220573

**Published:** 2019-08-02

**Authors:** Danielle R. Bruns, Philip D. Tatman, Roshni S. Kalkur, R. Dale Brown, Kurt R. Stenmark, Peter M. Buttrick, Lori A. Walker

**Affiliations:** 1 University of Colorado-Denver, Dept. of Medicine/Cardiology, Aurora, CO, United States of America; 2 Medical Scientist Training Program, Aurora, CO, United States of America; 3 University of Colorado-Denver, Dept. of Pediatrics, Aurora, CO, United States of America; University of Cincinnati College of Medicine, UNITED STATES

## Abstract

**Rationale:**

In virtually all models of heart failure, prognosis is determined by right ventricular (RV) function; thus, understanding the cellular mechanisms contributing to RV dysfunction is critical. Whole organ remodeling is associated with cell-specific changes, including cardiomyocyte dedifferentiation and activation of cardiac fibroblasts (Cfib) which in turn is linked to disorganization of cytoskeletal proteins and loss of sarcomeric structures. However, how these cellular changes contribute to RV function remains unknown. We’ve previously shown significant organ-level RV dysfunction in a large animal model of pulmonary hypertension (PH) which was not mirrored by reduced function of isolated cardiomyocytes. We hypothesized that factors produced by the endogenous Cfib contribute to global RV dysfunction by generating a heterogeneous cellular environment populated by dedifferentiated cells.

**Objective:**

To determine the effect of Cfib conditioned media (CM) from the PH calf (PH-CM) on adult rat ventricular myocytes (ARVM) in culture.

**Methods and results:**

Brief exposure (<2 days) to PH-CM results in rapid, marked dedifferentiation of ARVM to a neonatal-like phenotype exhibiting spontaneous contractile behavior. Dedifferentiated cells maintain viability for over 30 days with continued expression of cardiomyocyte proteins including TnI and α-actinin yet exhibit myofibroblast characteristics including expression of α-smooth muscle actin. Using a bioinformatics approach to identify factor(s) that contribute to dedifferentiation, we found activation of the PH Cfib results in a unique transcriptome correlating with factors both in the secretome and with activated pathways in the dedifferentiated myocyte. Further, we identified upregulation of periostin in the Cfib and CM, and demonstrate that periostin is sufficient to drive cardiomyocyte dedifferentiation.

**Conclusions:**

These data suggest that paracrine factor(s) released by Cfib from the PH calf signal a phenotypic transformation in a population of cardiomyocytes that likely contributes to RV dysfunction. Therapies targeting this process, such as inhibition of periostin, have the potential to prevent RV dysfunction.

## Introduction

In a number of animal models, inflammation contributes to the progression of left ventricular heart failure (HF) and in patients with left ventricular (LV) failure, the severity of HF correlates with plasma levels of inflammatory cytokines [1, 2]. In some studies, a direct causal link between LV dysfunction, LV remodeling and expression of pro-inflammatory cytokines, including TNF-α and IL-6, has been established [3, 4] and anti-inflammatory cytokines including interleukin 10 (IL10), thrombospondin-1, and TGF-β1 are reduced in patients with advanced heart failure [5–7]. Although prognosis in virtually all models of heart failure is determined by right ventricular (RV) function [8, 9], there is a relative paucity of biologic information about the cellular mechanisms that contribute to RV dysfunction. Similar to models of LV dysfunction [1, 2], recent information in models of pulmonary hypertension [10, 11] has suggested that biomarkers related to both inflammation and fibrosis, including TGF-β and IL-6 [3, 4], identify those at highest risk for RV deterioration, though how this pro-inflammatory environment leads to muscle deterioration has not been established.

Our group has a longstanding interest in a large animal model of PH and RV pressure overload–the bovine hypobaric hypoxia (HH) model. The HH model demonstrates significant resonance with human disease, and in particular, displays progressive deterioration of RV function [12]. As such it provides a nice context within which to study biologic factors that drive progressive RV dysfunction. However, in this model, isolated single cell mechanics show that individual cardiomyocytes have preserved (or even enhanced) contractility [13] despite whole organ level dysfunction, suggesting that a reductionist approach is inadequate to capture the cellular processes involved in the progressive RV dysfunction. Consistent with this, it has previously been shown that cardiac remodeling, associated with the focal loss of sarcomeric structure and heterogeneous disorganization of cardiac architecture, contributes to the pathogenesis of contractile dysfunction in several cardiac pathologies [14, 15], including in the bovine model of PH [12]. Remodeling is often accompanied by changes in cardiomyocyte expression of sarcomeric and cytoskeletal proteins, resulting in a cellular phenotype resembling the embryonic or neonatal state, with re-expression of the fetal gene program and isoform switching of multiple sarcomeric proteins [16]. These dedifferentiated myocytes, defined in part by their loss of sarcomeric structure and fetal gene expression, have been found in infarction border zones [17], the volume overloaded myocardium, and fibrillating atria [18]. While postulated to be initially protective against cardiac stress, continued activation of cardiomyocyte dedifferentiation is likely maladaptive [19].

In addition to myocyte disarray and dedifferentiation, cardiac remodeling and dysfunction also presents with changes in extracellular matrix composition, a process primarily regulated by cardiac fibroblasts (Cfib). In response to cardiac stress, fibroblasts undergo a phenotype conversion to myofibroblasts which is characterized by hyper-secretion of extracellular matrix components, expression of α-smooth muscle actin (α-SMA), and secretion of bioactive cytokines and growth factors [20]. In the bovine HH model, reprogramming of the fibroblast leads to phenotypic and functional remodeling of the distal pulmonary arterial wall. Together, the myocyte disarray, reduced mechanical coupling and activation of Cfib may all contribute to RV dysfunction.

The role dedifferentiation and myocyte disarray play in RV failure remains unexplored. Thus, we hypothesized that aberrant mechanical coupling of cardiac myocytes driven by expression of a dedifferentiated phenotype, contributes to the reduced ventricular function in this model. Furthermore, we hypothesized that local pro-inflammatory factors secreted by activated fibroblasts in vivo were responsible for the reduced mechanical coupling, resulting in reduced organ level performance.

## Methods

All animal use in this study was carried out in strict accordance with the recommendations in the Guide for the Care and Use of Laboratory Animals of the National Institutes of Health and was approved by the respective Institutional Animal Care and Use Committees at the University of Colorado-Denver (rodents) and Colorado State University (neonatal calves).

### Isolation of bovine cardiac tissues

The hypoxic hypobaric calf model of PH has been extensively characterized [21–24] and demonstrates significant RV pressure overload and RV dysfunction [13]. Briefly, neonatal calves were exposed to reduced atmospheric pressures for two weeks, during which time they develop marked PH. Control (CO) calves were housed at ambient Denver altitude. At the time of sacrifice, small 1 cm x 1 cm biopsies from the RV free wall were harvested and either snap frozen, embedded in optimal cutting temperature (OCT) embedding compound for future analysis or used to isolated right ventricular fibroblasts. RV fibroblasts were isolated following enzymatic digestion of bovine RV biopsies as previously described for human biopsies [25].

### Culture of bovine cardiac fibroblasts and generation of conditioned media

Following enzymatic digestion, RV fibroblasts were resuspended in Dulbecco’s modified eagle medium (DMEM) containing 10% fetal bovine serum and 1% streptomycin. Cells were plated and cultured under standard conditions of 37°C and 5% CO_2_. Once confluent, the fibroblasts were extensively washed with serum-free (SF) DMEM, and then were maintained in SF DMEM for 48 hours. After 48 hours, the conditioned media (CM) was collected for use. After collection of CM, fibroblasts were harvested for subsequent experimentation or passaged. For the current experiments, only low passage cells (p2-3) were used.

### Preparation of adult rat ventricular myocytes (ARVM)

Cardiac myocytes were isolated from healthy adult rats as previously described for mice [26]. Briefly, hearts were removed, hung on a Langendorff perfusion system and digested with collagenase at 37*°*. The ventricles were removed, minced and myocytes were liberated by gentle tituration. Cells were filtered through a 45 μm mesh and calcium was slowly reintroduced through a series of washes. Cells were resuspended in ACCT media (Medium 199 with Earle’s salts and 15 mM albumin, 2.5 mM carnitine, 5.8 mM creatinine and 5.0 mM taurine) [27] containing 10% fetal calf serum, plated on glass coverslips or cell culture plates pre-coated with laminin (10 μg/mL) and incubated at 37°C for two hours. After two hours, wells were rinsed with serum-free DMEM media and cells were maintained in the appropriate media (control serum-free DMEM or CM) for up to 45 days in a 5% CO_2_ incubator.

### Myocyte dedifferentiation

In some instances, dedifferentiation of cardiomyocytes was followed using grid-marked coverslips coated with laminin and imaged on an inverted microscope (Nikon TE-2000) with phase contrast objectives. Images were captured with a CCD Spot camera (Diagnostic Instruments) and morphologic analysis was conducted on a minimum of 10 cells/condition.

### qRT-PCR and immunoblotting

Myocytes, fibroblasts and ventricular tissues were harvested in Trizol for RNA isolation and reverse transcribed using iScript cDNA synthesis kit. Expression of the fetal gene program was assessed by qRT-PCR as previously described with 18S as a housekeeping gene [28]. Periostin expression was normalized to 18S and expressed relative to CO (Cfib) or freshly isolated ARVM (myocytes). Primer sequences for bovine and rat periostin are listed in Supporting Information (S1 Methods). Smooth muscle actin expression in hearts was normalized to 18S using bovine-specific primer sets (Qiagen). For western blot analysis of cardiomyocyte and Cfib protein expression, cells were washed with PBS, and proteins were solubilized in 8 M urea, 2.5 M thiourea, 4% CHAPS, 1 mM DTT and mixture of protease inhibitors. Protein expression was determined using SDS-PAGE and western blot analysis. For immunoblotting of conditioned media, proteins were precipitated by trichloroacetic acid, washed twice with cold acetone, and resuspended in Laemmli buffer for SDS-PAGE. Antibodies for immunoblotting are listed below.

### Immunocytochemistry in ARVM

The wells were rinsed with phosphate buffered saline (PBS) and fixed in 4% paraformaldehyde for 15 minutes. Cells were washed with PBS and incubated with pre-chilled (-20°C) methanol for 5 minutes. Cells were then rinsed with PBS, skinned in 1% Triton for 15 minutes, washed with PBS and blocked in 5% BSA for 2 hours. Cells were incubated in the appropriate antibody diluted in 1% BSA overnight at 4°C, rinsed in 1X PBS and incubated with fluorescent secondary antibody for one hour at room temperature to allow for visualization of the various proteins on the cardiac myocytes. DAPI staining was used to identify nuclei. The antibodies used for immunohistochemistry and immunoblotting were as follows: α-SMA: Abcam 5694; TnI: Phosphosolutions 2010; α-actinin: Sigma A7811; periostin: Santa Cruz sc-398631.

### Immunohistochemistry in RV sections

Immunohistochemistry was performed on a select subset of samples. Small biopsies of mid, free wall RV were embedded and frozen in OCT buffer. Frozen sections (8 μm) were cut transverse to the ventricular wall on a pre-cooled -20°C cryostat. Slides were washed in TBS containing 0.025% Tween-20 and incubated for 10 minutes in 1% Triton X-100 in phosphate buffered saline. Slides were washed, and blocked for two hours in 5% bovine serum albumin in TBS. Sections were incubated with primary antibody diluted in 1% BSA in TBS overnight. The sections were washed three times with TBS containing 0.025% triton, and incubated with secondary antibodies for one hour. Nuclei were visualized with DAPI. Sections were visualized on Zeiss Laser Scanning Microscope 780.

### Biochemical treatments of conditioned media

For experiments aimed at identification of active components of conditioned media, the media was subjected to fractionation or heat inactivation. Conditioned media was fractionated using Amicon Ultra centrifugal filter units with 30 kDa, 50 kDa or 100 kDa molecular weight cutoff (Sigma-Aldrich). For heat inactivation of complement, conditioned media was heated at 56°C for 30 min [29]. For depletion of periostin from the PH-CM, media was incubated with monoclonal periostin (Santa Cruz sc-398631, 0.2 μg/ml) for 1 hour with gentle rocking. Protein agarose A/G was added to pulldown the protein: antibody complex and was removed following centrifugation. The resulting conditioned media, was then used as periostin-depleted PH-CM.

### Mass spectrophotometric analysis of the secreted proteome

The fibroblast secretome was analyzed by mass spectrometry by MS Bioworks (Ann Arbor, MI). Proteins from either CO or PH-CM were precipitated with trichloroacetic acid (TCA) at 4°C and left overnight at -20°C. The resulting pellet was washed with 90% and 80% acetone. Acetone was removed and the material suspended in loading buffer. Protein concentration of the solubilized TCA pellet was determined by Qubit fluorometry (Invitrogen). 20μg of protein was separated by SDS-polyacrylamide gel electrophoresis and proteins were visualized by staining with InstantBlue Coomassie stain (Expedeon). Mobility regions were excised into forty equally sized bands based on a grid pattern. In-gel digestion with trypsin was performed on each excised band using a robot (ProGest, DigiLab) after reduction and alkylation. Each gel digest was analyzed by nano LC/MS/MS with a Waters NanoAcquity HPLC system interfaced to a ThermoFisher LTQ Orbitrap Velos Pro. Peptides were loaded on a trapping column and eluted over a 75μm analytical column at 350nL/min; both columns were packed with Jupiter Proteo resin (Phenomenex). The mass spectrometer was operated in data-dependent mode, with MS performed in the Orbitrap at 60,000 FWHM resolution and MS/MS performed in the LTQ. The fifteen most abundant ions were selected for MS/MS.

### RNA sequencing

RNA was isolated using standard Trizol protocol and ribosome-depleted using the Illumina TrueSeq kit. Libraries were sequenced on a HiSeq4000 by the Genomics and Microarray Core Shared Resource of the University of Colorado Cancer Center (P30CA046934).

### Bioinformatics

#### RNAseq

Fastq files from bovine fibroblasts were aligned to BosTau8, UCSC assembly. Fastq files from NRVMs were aligned to RN6, UCSC assembly. All fastq files were aligned using the HISAT2-STRINGTIE pipeline. Count matrices were loaded into the R statistical suite, and geometrically normalized using DEseq2. Genes that had an expression value greater than one in at least condition were considered for additional analysis. For quality control, principal components analysis was performed using R and to determine if treatment groups independently grouped. Genes were considered differentially expressed if they had P-value less than 0.05, calculated using a nonparametric, unequal variance, two-tailed t-test. The p-values were adjusted for multiple comparisons. Differentially expressed genes were log transformed, centered about their means, and clustered using Euclidean distance measurements with an average linkage k-means cluster. Pathway analysis was performed using GSEA software and databases supplied through the Msig database repository. Specifically, the Reactome, KEGG, Biocarta, GO molecular function, Hallmark, Onco Signatures, and Transcription factor databases were used. Human orthologues of bovine genes and proteins were identified and used for pathway enrichment. The RNASeq data for both the bovine cardiac fibroblasts and the dedifferentiated myocytes has been deposited in the Gene Expression Omnibus (GEO), accession number GSE133904.

### Statistical analysis

A students t-test was used to compare dedifferentiated and control myocytes for qRT-PCR gene expression, as well as between CO and PH fibroblasts. Statistical significance was set *a priori* at p<0.05.

## Results

### Pulmonary hypertension causes myocyte disarray and increased expression of α-smooth muscle actin expression in the right ventricle

**Fig 1:**
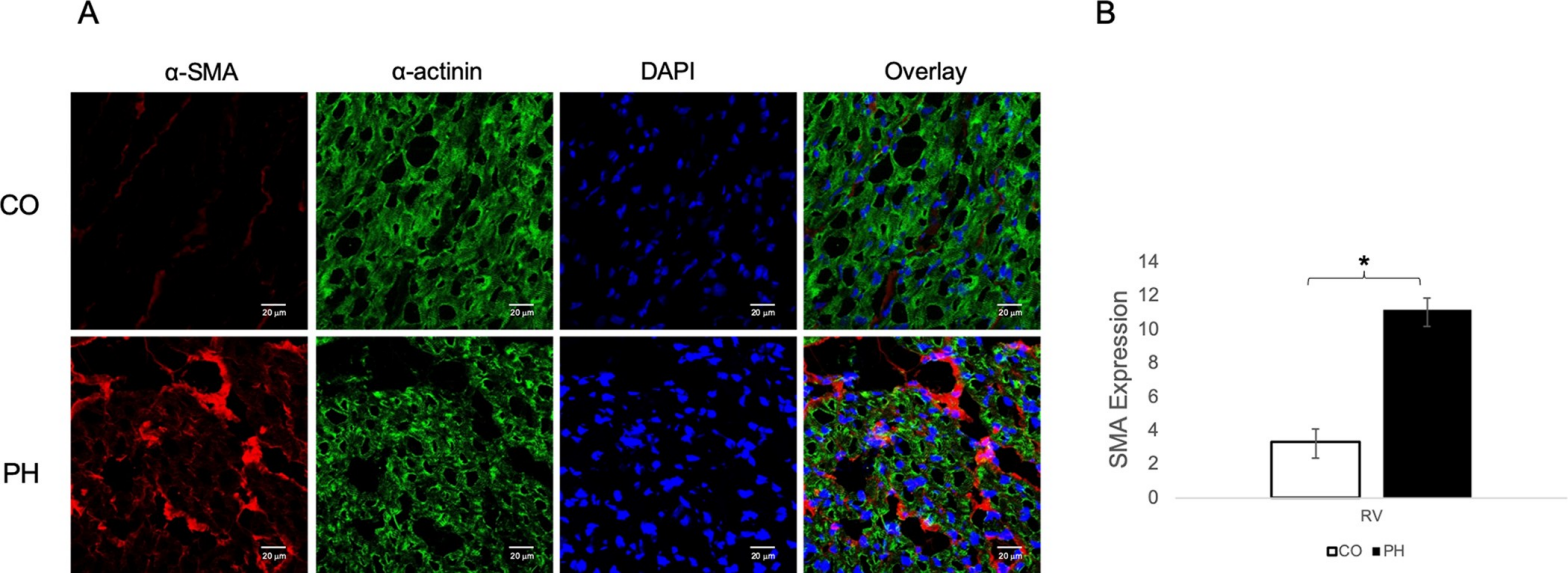
Pulmonary hypertension (PH) induces myocyte disarray in vivo. A) Representative images of mid-RV sections stained for α-SMA (red) and α-actinin (green). RV from control (CO) calves express little α-SMA and are highly organized. However, RV from PH-exposed (PH) calves express higher α-SMA and the normal organized structure of cardiac tissue is disrupted in areas expressing high α-SMA. DAPI (blue) identifies nuclei. Images were taken at 40X, with Zoom at 63X on a Zeiss Laser Scanning Microscope 780. B) Quantification of α-SMA in RV of control and PH-exposed calves (n = 12 CO; n = 12 PH).

The neonatal calf model of pulmonary hypertension-evoked RV dysfunction has been previously well-characterized phenotypically [12, 13]. To assess in vivo myocyte disarray and sarcomeric disruption, RV biopsies isolated from control calves (Denver altitude, CO) or calves with pulmonary hypertension (PH) were sectioned and stained with antibodies against cardiac α-actinin and α-SMA. The PH RV demonstrates significant expression of α-SMA compared to CO RV. Quantification of α-SMA by RT-PCR shows chamber-specific increases in the PH-RV compared to both control RV (Fig 1). In addition, staining of cardiac α-actinin in the PH RV is highly disorganized, with disruption of cardiac sarcomeric structure and expression of α-SMA in and around the myocytes (Fig 1).

### Pulmonary hypertension fibroblast conditioned media drives cardiomyocyte dedifferentiation

**Fig 2:**
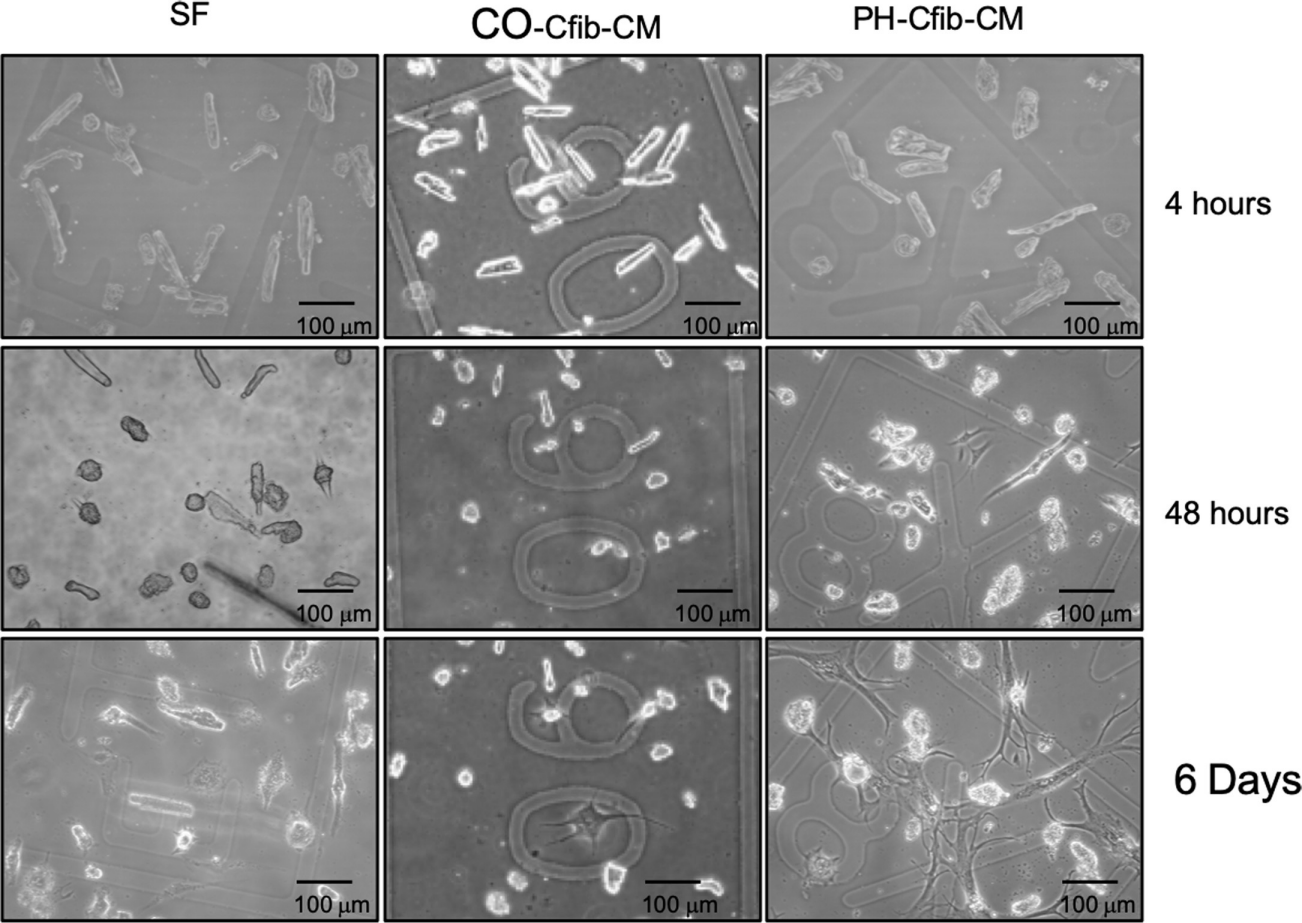
Cardiac fibroblast conditioned media (CM) from calves with pulmonary hypertension (PH) induced right ventricular dysfunction stimulates myocyte dedifferentiation. Adult rat ventricular myocytes (ARVM) were plated on etched coverslips to allow visual tracking of the same cell over time and incubated with serum-free (SF) media or CM derived from cardiac fibroblasts from calves exposed to control (CO) or PH conditions. By 48 hours myocytes in PH-Cfib-CM show signs of flattening, loss of striations, and branching of cellular processes. CM generated from control calf fibroblasts did not induce significant dedifferentiation.

To determine if local factors *in vivo* were driving sarcomeric disarray and inhibiting cardiomyocyte contractility (resulting in reduced organ-level performance), we generated conditioned media (CM) from cardiac fibroblasts derived from the RV of PH and CO calves. Adult rat ventricular myocytes (ARVM) were incubated with media from calves with PH and RV dysfunction (PH-CM), control calves (CO-CM) or SF DMEM media. Incubation of ARVM with PH-CM resulted in a dramatic, rapid dedifferentiation of the adult myocytes (Fig 2). The dedifferentiated myocytes lose their striations, flatten, and begin to beat autonomously within 4–5 days (S1 and S2 videos). Incubation of ARVM with SF DMEM resulted in a small phenotypic change in the myocyte, but did not demonstrate the same degree of dedifferentiation (Fig 2), and indeed when maintained in SF media, myocytes were only viable for approximately 5 days. To confirm that dedifferentiation is in fact stimulated by factors related to PH and not conditioned media per se, we incubated ARVM with CM generated from calves exposed to ambient Denver (control) atmosphere (CO-CM, Fig 2). Similar to SF DMEM, these cells do not demonstrate the same degree of dedifferentiation as PH-CM and are only viable for approximately 5–9 days. Since CO-CM and SF media both elicited similar phenotypes on dedifferentiation, subsequent experiments were only conducted in SF and PH-CM.

**Fig 3:**
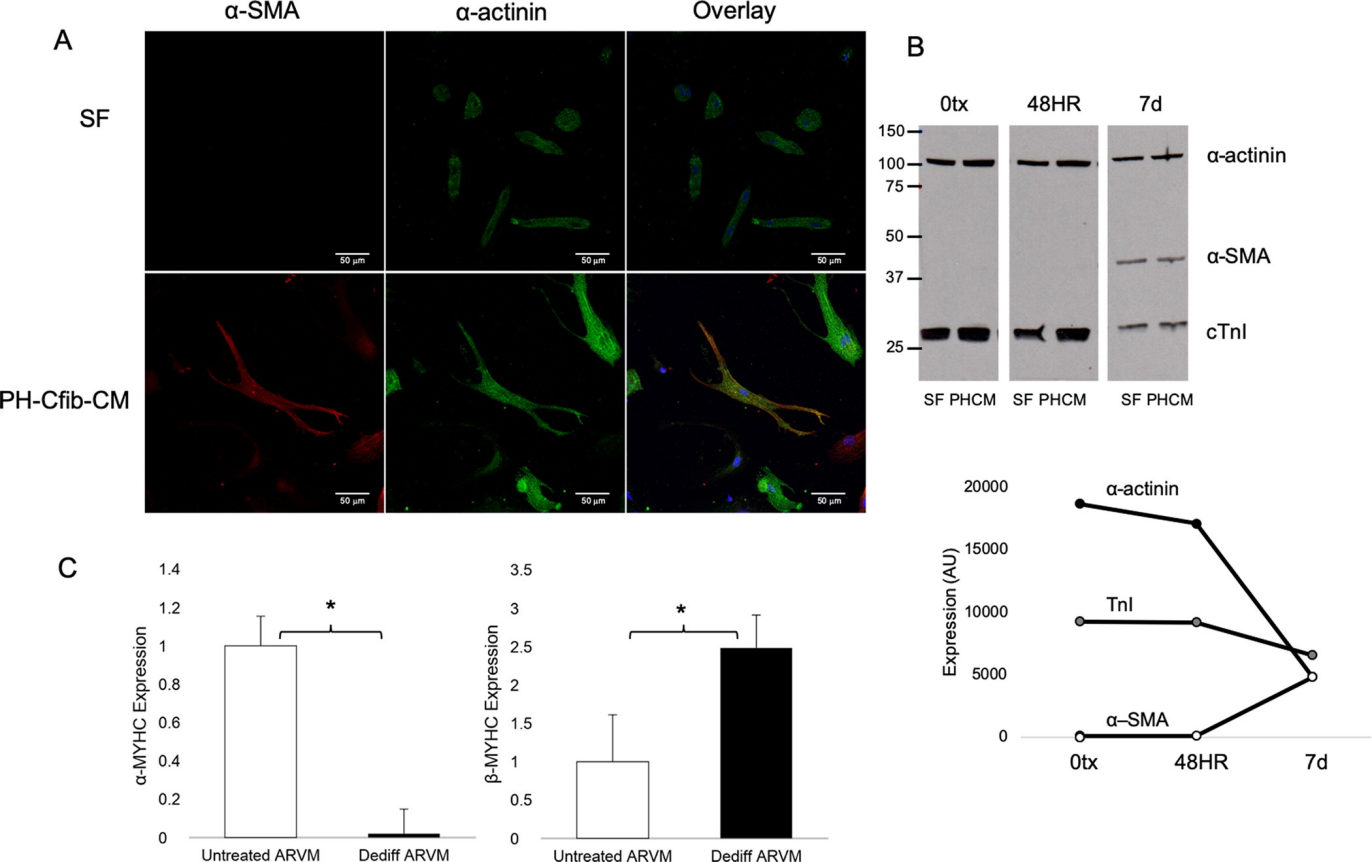
Dedifferentiated adult myocytes retain expression of cardiac markers. A) Immunofluorescent staining of dedifferentiated myocytes after 7 days SF or PH-CM exposure. Dedifferentiated cells still express α-actinin (green), while also starting to express α-SMA (red). Cells in SF media are less viable by 7 days, and do not dedifferentiate. Images were taken on a Zeiss Laser Scanning Microscope 780 at 40x objective. B) Protein expression of sarcomeric α-actinin, cardiac troponin I (cTnI) and α-SMA in myocytes exposed to SF media or PH-CM for 48 hours or 7 days. CD) Dedifferentiated myocytes activate the fetal gene program. By 7 days in culture, cells exposed to PH-CM demonstrate downregulated SERCA and α-MYHC expression. Expression of the fetal gene program was assessed by qRT-PCR using validated primers. Data are expressed as ΔCt to allow comparison across all groups. Higher reflects lower expression. n = 2 at each time-point. *p<0.05 compared to 0tx.

Immunohistochemical staining of dedifferentiated cells demonstrates that even after 7 days the myocyte-derived cells continue to express the cardiac cell marker sarcomeric α-actinin (Fig 3). By 7 days in culture, they also start to co-express α-SMA. During this period, nearly all SF and CO cells die, therefore we did not perform biochemical assessments in these cells. Western blot analysis supports the continued expression of cardiac cell markers troponin-I and sarcomeric α-actinin in dedifferentiated cells. By 7 days in culture, cells cultured in PH-CM begin to express α-SMA (Fig 3B). We then assessed expression of α and β myosin heavy chain (MYHC) in the cultured ARVM. As expected in a dedifferentiated state, after 7 days in PH media, ARVM significantly downregulated α-MYHC and demonstrated an increase in β-MYHC expression (Fig 3C and 3D).

### Cardiac fibroblast transcriptome and secretome analysis

To elucidate the factors responsible for dedifferentiation, we performed a series of partial purification experiments aimed at identifying biologically active components of the conditioned media. Crude fractionation using 50 kDa centrifugal filters resulted in fractions with partial biologic activity and fractionation with a 100 kDa centrifugal filter retained nearly all activity in the <100kDa fraction. Fractionation with a 30 kDa centrifugal filter resulted in activity that was maintained mainly in the >30kDa retentate, whereas the <30kDa flow through demonstrated minimal activity and most ARVM died within 7 days. (S1A Fig). Heat inactivation of conditioned media at 56° for 30 minutes largely deactivated the conditioned media, and ARVM exposed to heat inactivated media died within 7 days (S1B Fig). Together, these data suggest that the factors stimulating ARVM dedifferentiation are protein factors with a molecular mass of between 30kDa and 100kDa.

**Fig 4:**
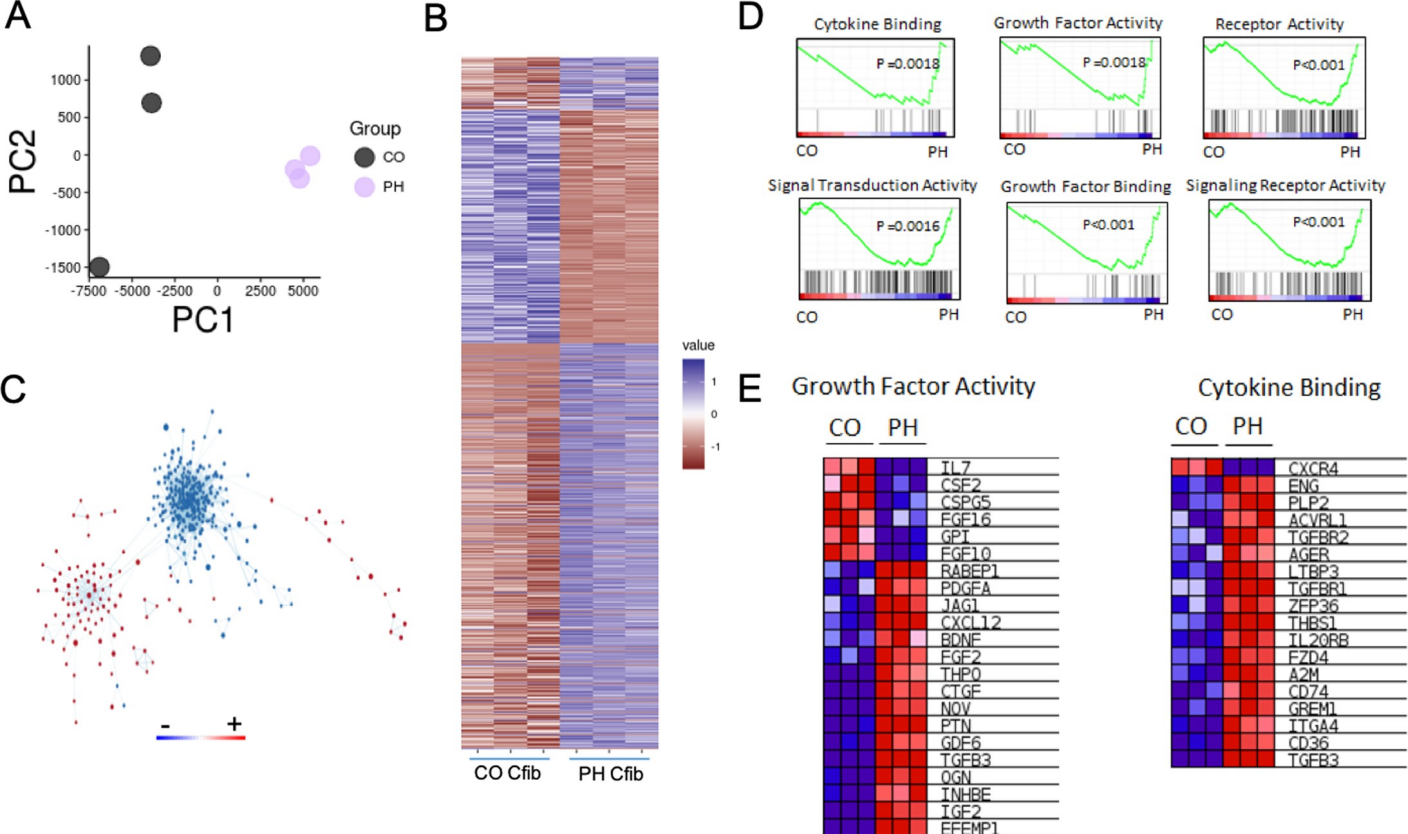
RNAseq analysis of control (CO) and pulmonary hypertension (PH) cardiac fibroblasts. A) PCA analysis of RNAseq data from CO and PH fibroblasts. B) Heatmap of gene expression comparing control fibroblasts to pulmonary hypertension fibroblasts. Genes with a significant difference of 0.05> were selected. Expression values were scaled from -1 to 1, similarity was calculated using an euclidean distance and clustered using a centroid linkage k-means method. C) Network of transcriptional changes comparing CO and PH fibroblasts. We displayed differentially enriched pathways from GSEA as enrichment networks, in which each node is a pathway, and each link between nodes is a common gene. The size of the nodes indicates the number of genes found in our data that are in the pathway. Red coloring indicates pathways enriched in the PH group, whereas blue indicates pathways enriched in the CO group. The CO and PH networks are independent, suggesting significant divergence of in the signaling cascades between these two groups of cells. D) Select enrichment plots highlighting the enriched pathways identified by GSEA. E) Two representative heatmaps for the Growth Factor Activity and Cytokine Binding pathways showing the genes responsible for the enrichment.

In an effort to identify the relevant dedifferentiation factors we undertook a bioinformatics approach to look at global changes in the fibroblast transcriptome. We performed RNAseq on fibroblasts from three animals from each group (S1 Table). As seen in Fig 4A, principal component analysis (PCA) demonstrates separation of PH and CO fibroblasts, indicating significant differences in the transcriptomes between these groups. In total, 2115 genes were found to be either significantly increased or decreased in expression (Fig 4B). GSEA analysis revealed independent transcriptional networks (Fig 4C). Analysis of the differential pathways by both log(p) and the normalized enrichment scores revealed significant activation of secretory pathways, extracellular matrix manipulation, hypoxia, and the production of numerous ligands (S2–S7 Figs). Consistent with our hypothesis that dedifferentiation is being driven by the generation of signaling molecules, there was significant enrichment of the GO molecular Function pathway for growth factor activity, receptor binding activity, signal transduction and cytokine binding (Fig 4D and 4E). Together these data identify a unique transcriptome profile in the PH Cfib that is associated with message for signaling cytokines, growth factors and extracellular matrix proteins. Since the PH-CM transcriptome was largely enriched for secretory cytokines, growth factors and extracellular matrix proteins, we sought to analyze the unique components PH-CM.

**Fig 5:**
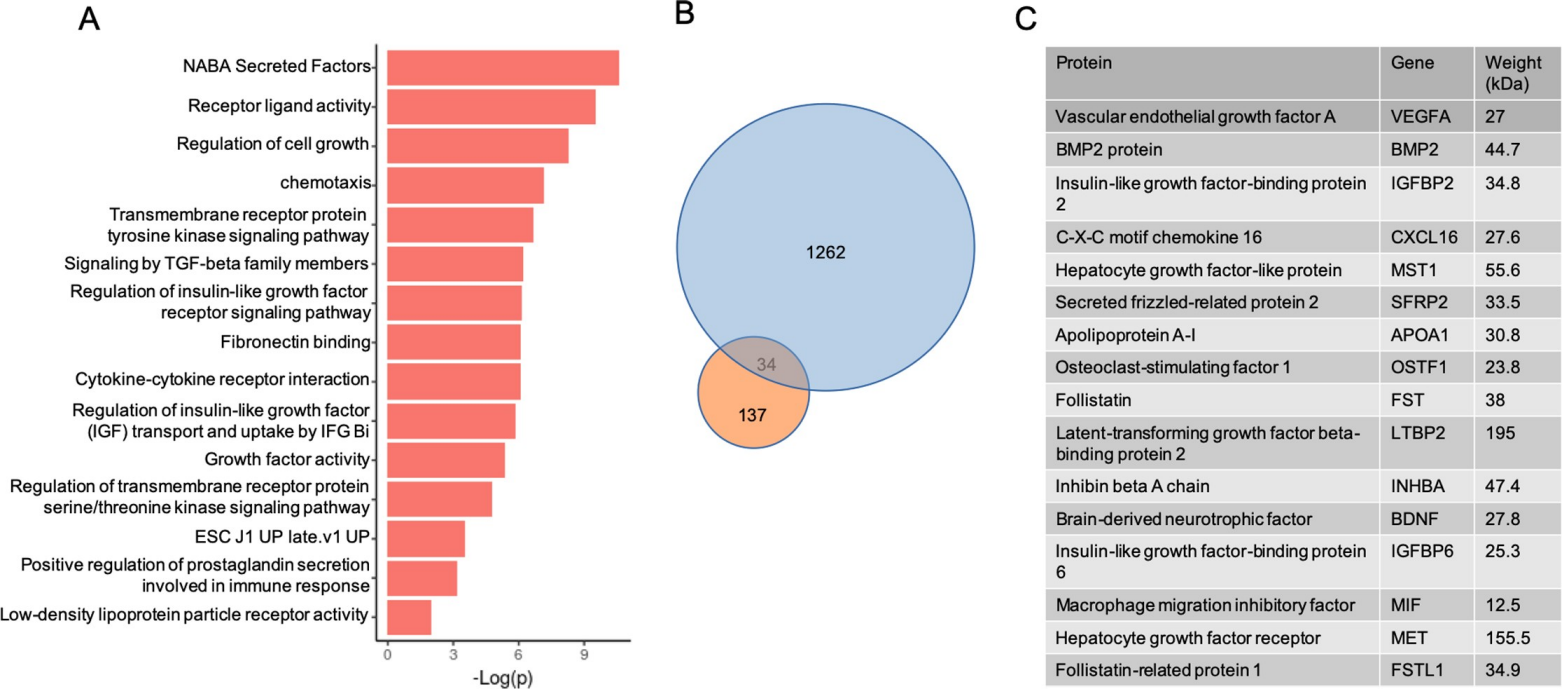
Conditioned media pathway analysis of the extracellular signaling molecules. A) Pathway analysis of signaling molecules upregulated in the PH-CM. B) Comparison of the upregulated genes in the PH Cfib transcriptome (blue circle) with significantly enhanced proteins in the PH-CM (orange circle). Of the 137 secreted proteins found enriched in the conditioned media, the transcripts of 34 of these were also found increased in the transcriptome of the PH fibroblasts. C) Representative list of 16 of the 34 proteins identified as enriched in the PH conditioned media with known signaling properties that were also found upregulated in the PH Cfib transcriptome. The human orthologues are displayed in the table and were then used for pathway analysis.

To identify the secreted factors generated by the PH fibroblast, we analyzed the conditioned media by mass spec (S2 Table). We considered proteins that had a greater than two-fold enrichment in the PH conditioned media to be significant, resulting in 137 proteins. Pathway analysis of the 137 protein cohort identified enrichment of extracellular matrix reorganizing pathways, IGF1R signaling, tyrosine kinase receptor signaling, and cellular morphogenesis and differentiation pathways (Fig 5A). The transcripts of 34 of these proteins were also found to be increased in RNAseq data (Fig 5B) and we were able to identify 17 enriched proteins that have known ligand activity (seven of which are between 30–90 kDA). Pathway analysis of these proteins found a similar pathway enrichment profile as the full cohort of the full 137 gene cohort. Specifically, tyrosine kinase receptor binding, IGF1 signaling, cell growth, and stem cell signaling were all significantly enriched (Fig 5C). These data support the hypothesis that enhanced survival of the ARVMs treated in culture is due to secreted proteins that originate from PH fibroblasts, proteins which have growth and stem cell-like signaling properties.

### RNAseq in dedifferentiated adult rat ventricular myocytes

**Fig 6:**
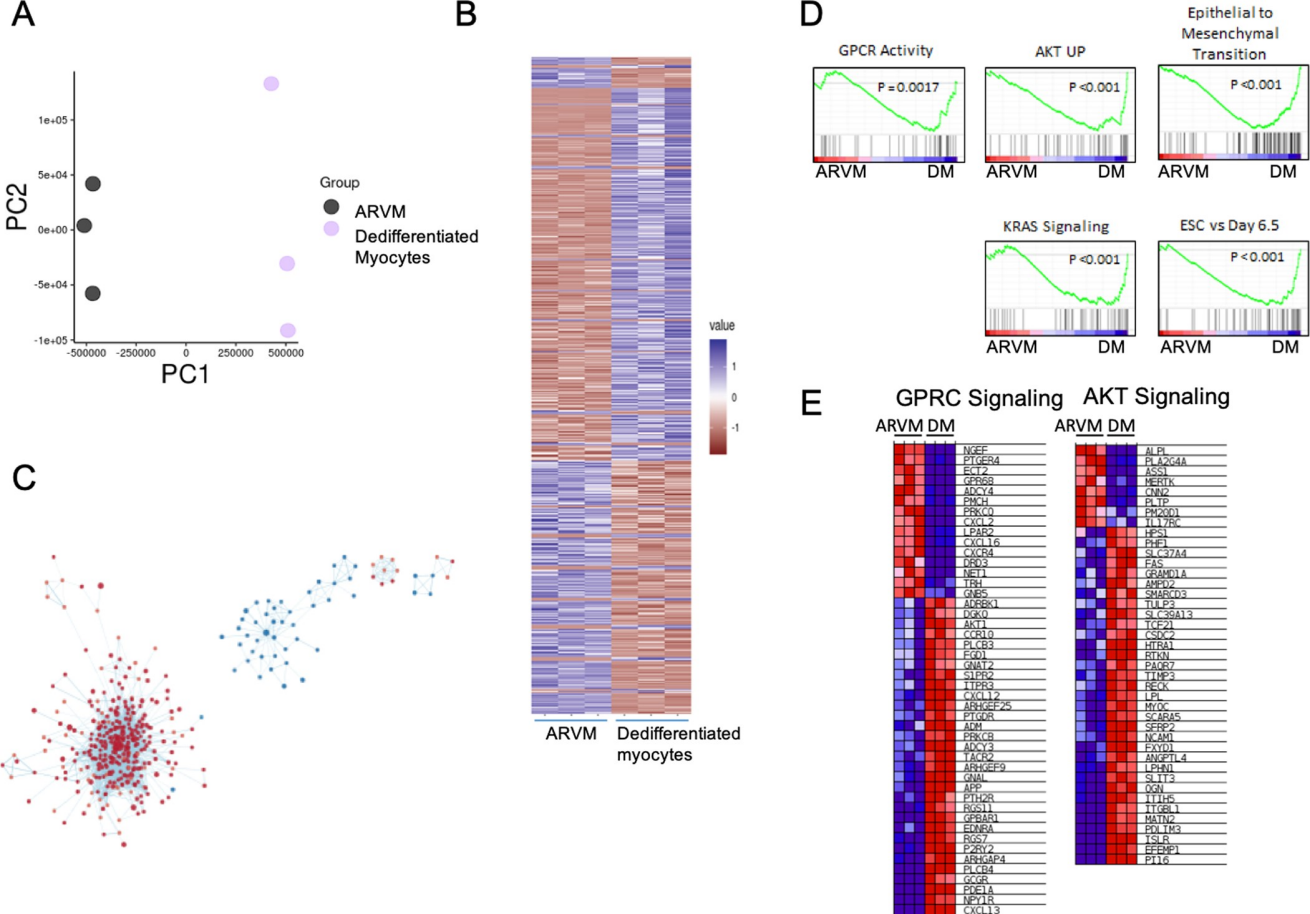
Dedifferentiated adult ventricular myocyte (ARVM) RNAseq. A) PCA plot of ARVMs treated with and without conditioned media from PH fibroblasts. B) Heatmap of gene expression comparing control, freshly isolated ARVMs to ARVMs treated with PH Fibroblast conditioned media. Genes with a significant difference of <0.05 were selected. Expression values were scaled from -1 to 1, similarity was calculated using an euclidean distance and clustered using a centroid linkage k-means method. C) Network of transcriptional changes. We displayed differentially enriched pathways from GSEA as enrichment networks in which each node is a pathway, and each link between nodes is a common gene. The size of the nodes indicates the number of genes found in our data that are in the pathway. Red coloring indicates pathways enriched in the ARVM PH group, whereas blue indicates pathways enriched in the control ARVM group. D) Select enrichment plots highlighting the enriched pathways identified by GSEA. E) Representative heat maps for G-protein coupled receptor, downstream signaling pathways (GPCR signaling) and AKT signaling pathways showing the genes responsible for enrichment.

To identify the pathways activated in the ARVM that result in dedifferentiation, we analyzed the transcriptomes of PH-CM treated ARVMs and untreated, freshly isolated ARVMs by RNAseq (S3 Table). The treatment groups clustered by PCA (Fig 6A), suggesting global significant differences in the transcriptomes of freshly isolated ARVM and the dedifferentiated ARVM. We were able to identify 7052 significantly differentially expressed genes (Fig 6B). Pathway analysis by GSEA of these genes revealed enrichment of cell cycle, extracellular protein pathways, TGFβ signaling, WNT signaling, Kras signaling, and tyrosine kinase receptor signaling (S8–S13 Figs). Consistent with our hypothesis that dedifferentiation is being driven by the fibroblast generation of signaling molecules, there was significant enrichment of the GO molecular Function pathway for GPCR Activity and AKT signaling upregulation (Fig 6D and 6E). Epithelial to mesenchymal transition (EMT) signaling was also significantly enriched in the dedifferentiated ARVM.

### Identification of periostin as a driver of adult myocyte dedifferentiation

**Fig 7:**
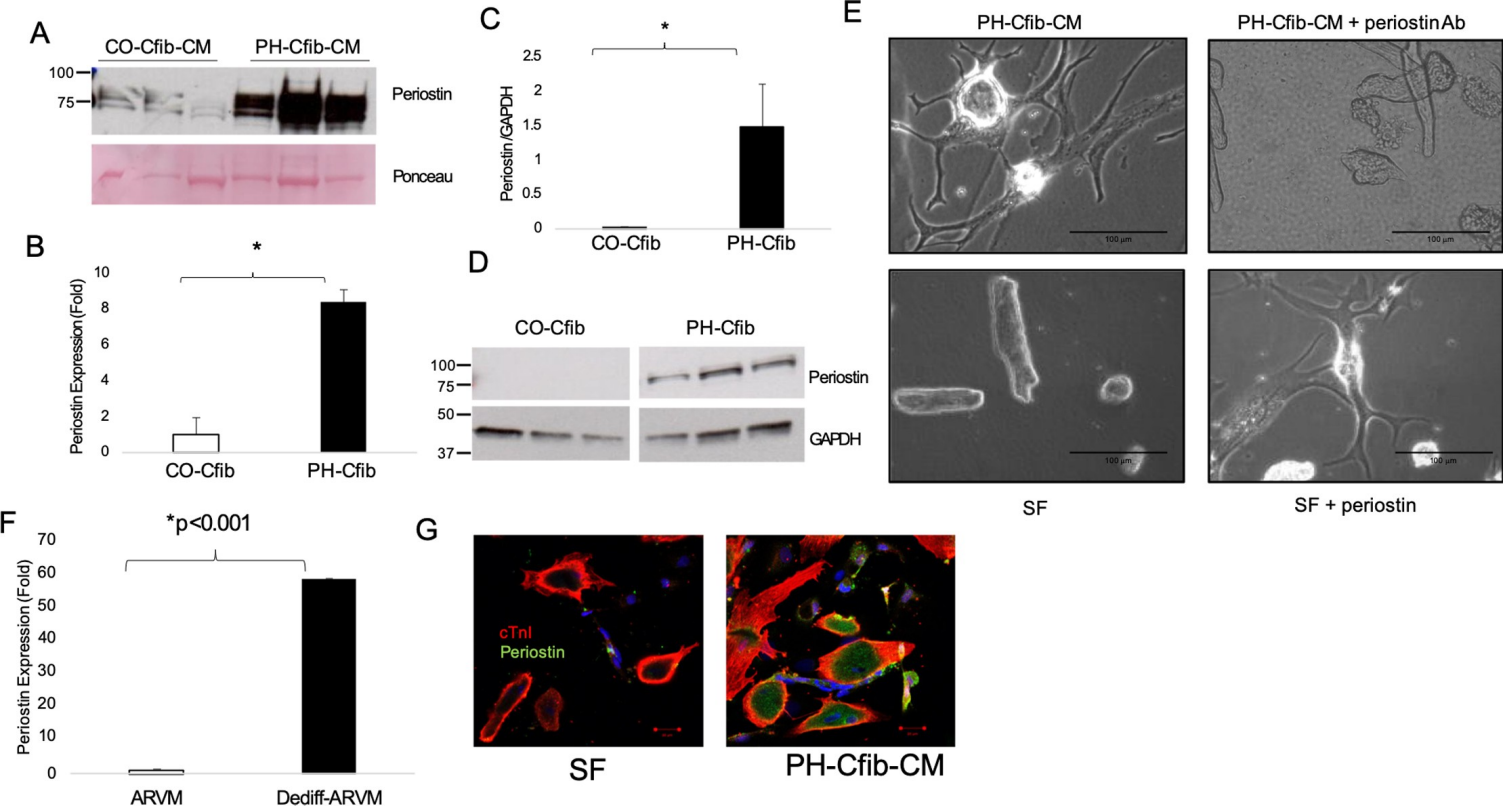
Periostin drives adult myocyte dedifferentiation. A) Periostin is significantly expressed in the PH-CM compared to CO-CM. B) Periostin RNA and C,D) protein expression are significantly higher in PH Cfib compared to CO. E) Immunodepletion of periostin from the PH-CM attenuates dedifferentiation, while addition of recombinant periostin to SF media stimulates dedifferentiation. F) Periostin expression is robustly upregulated in the dedifferentiated myocytes compared to freshly isolated ARVM. G) Periostin protein expression is upregulated in dedifferentiated cells compared to SF-treated. Periostin RNA and protein expression were assessed by qRT-PCR and immunoblotting, respectively. Experiments were performed in 3 PH and 2 CO calves in biological triplicate. *p<0.05 by student’s t-test.

In an effort to identify putative regulators of dedifferentiation, we analyzed the secretome as well as RNA-seq from both the Cfib and the dedifferentiated myocytes to find commonly expressed targets across all three analyses. One of the top proteins in the Cfib secretome and Cfib RNA-seq was periostin. We confirmed periostin expression in the conditioned media by immunoblotting and found it to be significantly enriched in the PH media compared to CO (Fig 7A). Further, Cfib from the PH calves express significantly higher periostin at the transcriptional (Fig 7B) and protein levels (Fig 7C and 7D). To test the requirement of periostin for PH-CM mediated dedifferentiation, we depleted periostin from the PH-CM and found significant blunting of the dedifferentiation phenotype (Fig 7E). Similarly, addition of recombinant periostin to SF media robustly induced dedifferentiation, apparent within 4 days of treatment (Fig 7E). We then identified periostin in the dedifferentiated RNA-seq analysis, which we confirmed by qRT-PCR to be nearly 60-fold upregulated in the dedifferentiated myocytes compared to control (Fig 7F). Periostin was also expressed at the protein level in PH-CM treated cells while absent in the SF-treated, as assessed by immunocytochemistry (Fig 7G).

## Discussion

**Fig 8:**
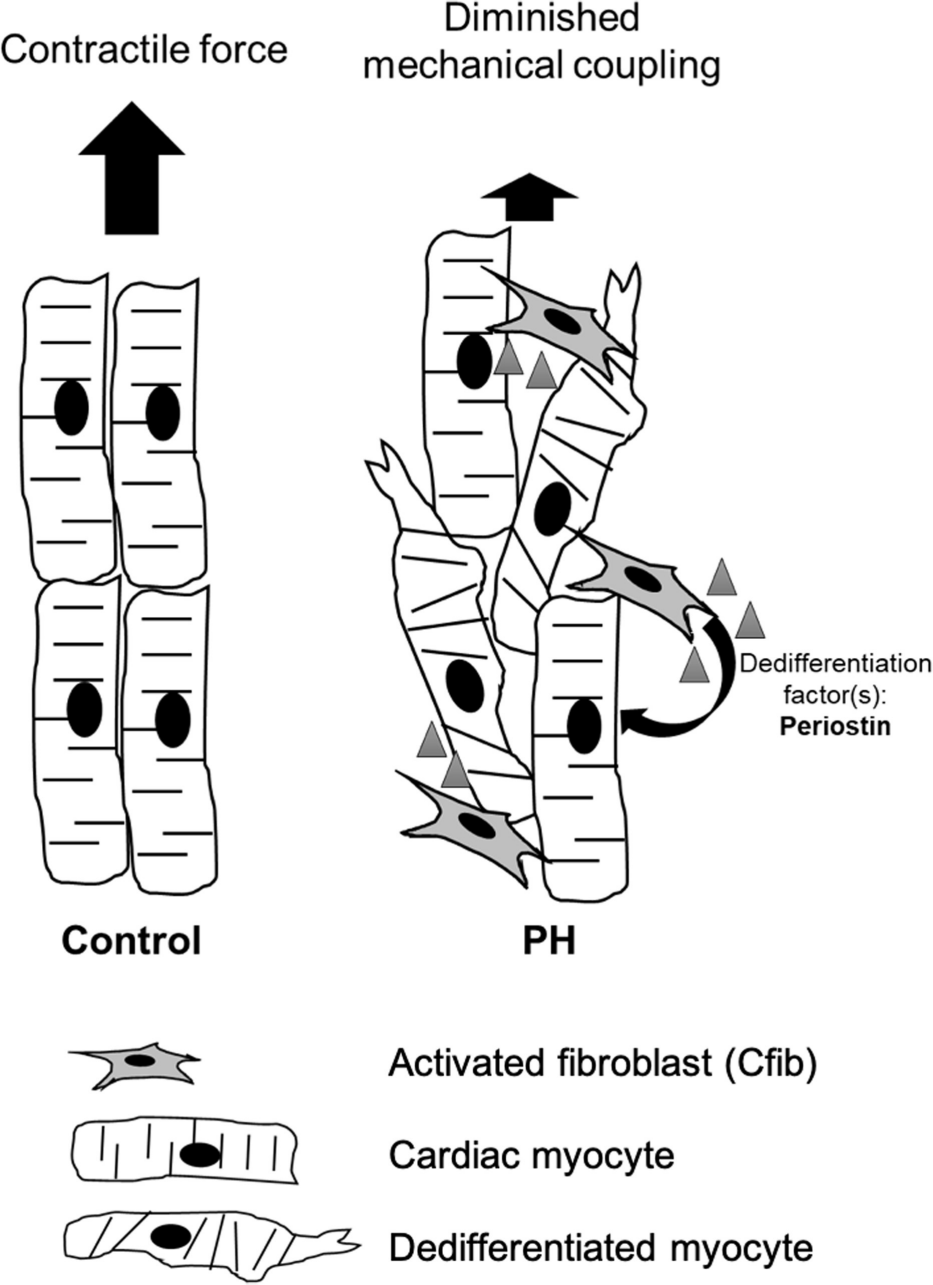
Working model of Cfib-mediated disruption of sarcomeric organization and loss of contractile function. Identification of additional secreted factor(s) which drive dedifferentiation and myocyte plasticity will improve therapeutic strategies.

Pulmonary hypertension-induced right heart failure is a devastating disease with poor prognosis. Loss of sarcomeric structure and disorganization of cardiac architecture contributes to the pathogenesis of contractile dysfunction in several cardiac pathologies. Thus, we tested the hypothesis that local factors released by activated RV cardiac fibroblasts resulted in dedifferentiation of ARVM and sarcomeric disarray, which in vivo would lead to reduced contractility and reduced organ-level performance. Here, we show that treatment of ARVM with conditioned media from PH cardiac fibroblasts causes marked and rapid dedifferentiation, an effect mediated through secretion of protein factor(s) from the activated fibroblasts, most likely periostin. Both the dedifferentiated myocytes and the activated fibroblasts then contribute to ventricular and sarcomeric disarray and poor contractile performance (Fig 8), through sarcomeric disarray and extracellular matrix deposition respectively.

Our data demonstrate that Cfib PH-CM stimulates dedifferentiation of adult cardiac myocytes to a phenotypically neonatal-like-cell. This is of significant interest as dedifferentiation is a requisite for driving adult cardiomyocytes into a proliferative state for therapeutic repair of the heart. Despite the growing interest in the dedifferentiation of adult cardiomyocytes, the factors which drive dedifferentiation have remained elusive. Further, the physiological or biological impact of dedifferentiation on the remodeling heart remains unclear, though some recent data suggests that though dedifferentiation initially protects the stressed heart, chronically it fails to support cardiac structure and function [19]. While we did not directly assess the contractile impact of dedifferentiation, we hypothesize that dedifferentiated cardiomyocytes exhibit significantly reduced force and therefore contribute dramatically to organ level dysfunction.

Though efforts are still underway in our lab to comprehensively identify all factor(s) responsible for dedifferentiation, we did identify periostin as a significant regulator of this process. Periostin, a 90kDa secreted extracellular matrix protein, is critical for determination of cell fate, proliferation and migration of cancer cells [30, 31], and has been implicated in inflammatory disease including cardiovascular disease [32]. While periostin expression is nearly undetectable in the healthy heart, it is robustly upregulated in response to pathological stress [33–35], with expression thought to be primarily located in the activated myofibroblasts [36–38]. Genetic loss of periostin function from activated fibroblasts attenuates fibrotic remodeling in response to LV pressure overload [39], and delivery of a periostin neutralizing antibody improves post-myocardial infarction remodeling [40]. Here, we report robust upregulation of periostin in RV fibroblasts derived from the PH calf, and secretion of periostin from these activated fibroblasts. These data highlight for the first time a role for periostin in pulmonary hypertension and RV dysfunction and suggest that strategies which interrupt Cfib-mediated expression and secretion of periostin may be therapeutically viable in this disease.

Conversely, the role for periostin in non-fibroblast cell populations remains unresolved. In 2008, Kuhn et al. observed that periostin induced cell cycle reentry in ARVM and that administration of periostin improved cardiac function through stimulation of cardiac regeneration [41]. However, this observation was quickly followed by a report in which periostin did not induce adult cardiomyocyte proliferation using a periostin α-MYHC promoter [42], generating a debate as to whether periostin is truly regenerative. Here, we report that PH-CM induces rapid and robust dedifferentiation, an effect which is attenuated when periostin is removed from the media. Further, administration of recombinant periostin causes rapid and robust dedifferentiation of ARVM. Together, these data strongly support periostin as a mediator of adult myocyte dedifferentiation. Understanding the mechanisms of periostin-mediated dedifferentiation, downstream periostin signaling, and identifying small molecular mediators of this pathway is therefore necessary to harness the therapeutic potential of periostin. Importantly, we speculate that both activation and inhibition of periostin signaling may have therapeutic potential depending on the nature of cardiac insult and the timing of therapeutic intervention.

While our data demonstrate a significant role for periostin in mediating cellular and ventricular changes to hypoxia-induced pressure overload, it is very likely that other mediators are involved in the *in-vivo* adaptation to this insult. It is increasingly recognized that exosomes, small extracellular vesicles carrying a variety of bioactive molecules can regulate cellular performance [43] and exosomes have been implicated in the pathogenesis of cardiac disease. Exosomal packaging of proteins such as HSP60 [44], and of microRNAs [45] has been shown to mediate paracrine signaling between cardiac fibroblasts and cardiac myocytes. Further study is necessary to determine whether exosomes mediate cellular responses in our model.

In conclusion, our data demonstrate that hypobaric hypoxia and RV pressure overload results in activation of the cardiac fibroblasts, secretion of growth factors, cytokines and a variety of other signaling pathways. Importantly, we have been able to identify proteins in the PH Cfib secretome, encoded in the activated Cfib transcriptome, that resulted in activation of specific pathways in the dedifferentiated ARVM. Specifically, we demonstrate a role for Cfib-generated periostin in driving dedifferentiation and propose further work aimed at developing periostin focused therapies to improve fibrotic remodeling while also regulating myocyte dedifferentiation/regeneration.

## Supporting information

S1 Methods Primer sequences for bovine and rat periostin and 18S. Periostin expression was normalized to 18S and expressed relative to CO (Cfib) or freshly isolated ARVM (myocytes).
(PDF)

S1 Video Dedifferentiated myocytes beat autonomously in culture. Adult rat ventricular myocytes (ARVM) were exposed to fibroblast conditioned media from pulmonary hypertension (PH-CM) calves. Exposure of ARVM to serum-free or conditioned media from control calves does not cause robust dedifferentiation. Video was taken at 10 X magnification after 4 days of PH-CM exposure.
(MP4)

S2 Video Addition of 1% fetal calf serum to PH-CM allows cells to be maintained for 35 days during which the beating “islands” of dedifferentiated cells continue to grow, beat, and become more structured in appearance. Video was taken at 4X magnification after 26 days of PH-CM exposure.
(MP4)

S1 Fig A: Cardiac fibroblast conditioned media (CM) from calves with pulmonary hypertension (PH) was fractionated using a 30 kDa centrifugal filter. Retentate (>30 kDa) and filtrate (<30 kDa) volumes were normalized to starting volume and incubated with ARVM. Incubation with >30 kDa fraction resulted in significant dedifferentiation whereas the <30 kDa was associated with ARVM death. B: Heat inactivation of PH-CM. Heat-inactivated PH-CM was incubated with ARVM for up to 21 days and was associated with significantly reduced dedifferentiation and increased ARVM death. Bottom right; red–SMA staining.
(PDF)

S2 Fig Reactome pathway analysis of CO vs PH Fibroblasts using GSEA software from the Broad Institute. NES is the normalized enrichment score, which is shown as red bars, and the logP is displayed as either the logP or–logP to indicate enrichment or lack of enrichment in the PH group. Positive values indicate enrichment in the PH group. Negative values indicate a lack of enrichment in the PH group, or enrichment in the CO group.
(PDF)

S3 Fig Oncogenic Signature pathway analysis of CO vs PH Fibroblasts using GSEA software from the Broad Institute. NES is the normalized enrichment score, which is shown as red bars, and the logP is displayed as either the logP or–logP to indicate enrichment or lack of enrichment in the PH group. Positive values indicate enrichment in the PH group. Negative values indicate a lack of enrichment in the PH group, or enrichment in the CO group.
(PDF)

S4 Fig Hallmark pathway analysis of CO vs PH Fibroblasts using GSEA software from the Broad Institute. NES is the normalized enrichment score, which is shown as red bars, and the logP is displayed as either the logP or–logP to indicate enrichment or lack of enrichment in the PH group. Positive values indicate enrichment in the PH group. Negative values indicate a lack of enrichment in the PH group, or enrichment in the CO group.
(PDF)

S5 Fig GO MF pathway analysis of CO vs PH Fibroblasts using GSEA software from the Broad Institute. NES is the normalized enrichment score, which is shown as red bars, and the logP is displayed as either the logP or–logP to indicate enrichment or lack of enrichment in the PH group. Positive values indicate enrichment in the PH group. Negative values indicate a lack of enrichment in the PH group, or enrichment in the CO group.
(PDF)

S6 Fig Transcription Factor Expression profile analysis of CO vs PH Fibroblasts using GSEA software from the Broad Institute. NES is the normalized enrichment score, which is shown as red bars, and the logP is displayed as either the logP or–logP to indicate enrichment or lack of enrichment in the PH group. Positive values indicate enrichment in the PH group. Negative values indicate a lack of enrichment in the PH group, or enrichment in the CO group.
(PDF)

S7 Fig KEGG pathway analysis of CO vs PH Fibroblasts using GSEA software from the Broad Institute. NES is the normalized enrichment score, which is shown as red bars, and the logP is displayed as either the logP or–logP to indicate enrichment or lack of enrichment in the PH group. Positive values indicate enrichment in the PH group. Negative values indicate a lack of enrichment in the PH group, or enrichment in the CO group.
(PDF)

S8 Fig Biocarta pathway analysis of CO vs PH Fibroblasts using GSEA software from the Broad Institute. NES is the normalized enrichment score, which is shown as red bars, and the logP is displayed as either the logP or–logP to indicate enrichment or lack of enrichment in the PH group. Positive values indicate enrichment in the PH group. Negative values indicate a lack of enrichment in the PH group, or enrichment in the CO group.
(PDF)

S9 Fig GO Molecular Function pathway analysis of CO vs PH ARVMs using GSEA software from the Broad Institute. NES is the normalized enrichment score, which is shown as red bars, and the logP is displayed as either the logP or–logP to indicate enrichment or lack of enrichment in the PH group. Positive values indicate enrichment in the PH group. Negative values indicate a lack of enrichment in the PH group, or enrichment in the CO group.
(PDF)

S10 Fig Biocarta pathway analysis of CO vs PH ARVMs using GSEA software from the Broad Institute. NES is the normalized enrichment score, which is shown as red bars, and the logP is displayed as either the logP or–logP to indicate enrichment or lack of enrichment in the PH group. Positive values indicate enrichment in the PH group. Negative values indicate a lack of enrichment in the PH group, or enrichment in the CO group.
(PDF)

S11 Fig Onc Sig pathway analysis of CO vs PH ARVMs using GSEA software from the Broad Institute. NES is the normalized enrichment score, which is shown as red bars, and the logP is displayed as either the logP or–logP to indicate enrichment or lack of enrichment in the PH group. Positive values indicate enrichment in the PH group. Negative values indicate a lack of enrichment in the PH group, or enrichment in the CO group.
(PDF)

S12 Fig Hallmark pathway analysis of CO vs PH ARVMs using GSEA software from the Broad Institute. NES is the normalized enrichment score, which is shown as red bars, and the logP is displayed as either the logP or–logP to indicate enrichment or lack of enrichment in the PH group. Positive values indicate enrichment in the PH group. Negative values indicate a lack of enrichment in the PH group, or enrichment in the CO group.
(PDF)

S13 Fig Kegg pathway analysis of CO vs PH ARVMs using GSEA software from the Broad Institute. NES is the normalized enrichment score, which is shown as red bars, and the logP is displayed as either the logP or–logP to indicate enrichment or lack of enrichment in the PH group. Positive values indicate enrichment in the PH group. Negative values indicate a lack of enrichment in the PH group, or enrichment in the CO group.
(PDF)

S14 Fig Reactome pathway analysis of CO vs PH ARVMs using GSEA software from the Broad Institute. NES is the normalized enrichment score, which is shown as red bars, and the logP is displayed as either the logP or–logP to indicate enrichment or lack of enrichment in the PH group. Positive values indicate enrichment in the PH group. Negative values indicate a lack of enrichment in the PH group, or enrichment in the CO group.
(PDF)

S1 Table Final enriched genes from RNA-seq from RV cardiac fibroblasts. (XLSX)

S2 Table Final enriched proteins from RV fibroblast secretome. (XLSX)

S3 Table Final enriched genes from RNA-seq from dedifferentiated ARVM. (XLSX)

## Abbreviations

Cfib: cardiac fibroblast
ARVM: adult rat ventricular myocyte
PH: pulmonary hypertension
PH-CM: conditioned media generated from the calf with pulmonary hypertension
CO-CM: conditioned media generated from the control calf
HH: hypobaric hypoxia
