# Supplementary material for: The right ventricular fibroblast secretome drives cardiomyocyte dedifferentiation
Source: PLoS One. 2019 Aug 2;14(8):e0220573. doi: 10.1371/journal.pone.0220573 (PMC6677314; doi:10.1371/journal.pone.0220573)
Supplement: S1 Methods — Periostin expression was normalized to 18S and expressed relative to CO (Cfib) or freshly isolated ARVM (myocytes). (PDF) [file pone.0220573.s001.pdf]

Detailed Methods

The primer sequences are as follows:

Rat periostin Forward: 5'-AGGAGCCGTGTTTGAGACCAT-3'

Rat periostin Reverse: 5'-CGGTGAAAGTGGTTTGCTGTTT-3'

Bovine periostin Forward: 5'-ACTTCACACTCTTTGCTCCC-3'

Bovine periostin Reverse: 5'-CCATGATAGCCTCAGAACACTG-3'

18S Forward: 5'-GCCGCTAGAGGTGAAATTCTTG-3'

18S Reverse: 5'-CTTTCGCTCTGGTCCGTCTT-3'
