# Supplementary figures and images for: The right ventricular fibroblast secretome drives cardiomyocyte dedifferentiation
Source: PLoS One. 2019 Aug 2;14(8):e0220573. doi: 10.1371/journal.pone.0220573 (PMC6677314; doi:10.1371/journal.pone.0220573)

## Supplemental Figure 1

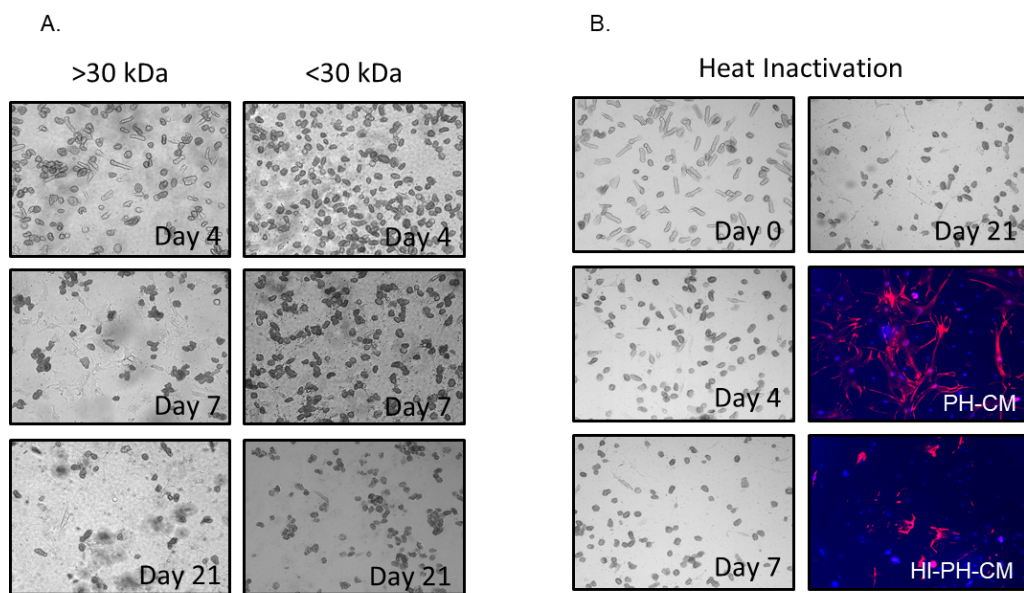

Supplement: S1 Fig — A: Cardiac fibroblast conditioned media (CM) from calves with pulmonary hypertension (PH) was fractionated using a 30 kDa centrifugal filter. Retentate (>30 kDa) and filtrate (<30 kDa) volumes were normalized to starting volume and incubated with ARVM. Incubation with >30 kDa fraction resulted in significant dedifferentiation whereas the <30 kDa was associated with ARVM death. B: Heat inactivation of PH-CM. Heat-inactivated PH-CM was incubated with ARVM for up to 21 days and was associated with significantly reduced dedifferentiation and increased ARVM death. Bottom right; red–SMA staining. (PDF) [file pone.0220573.s004.pdf]

Supplemental Figure 2

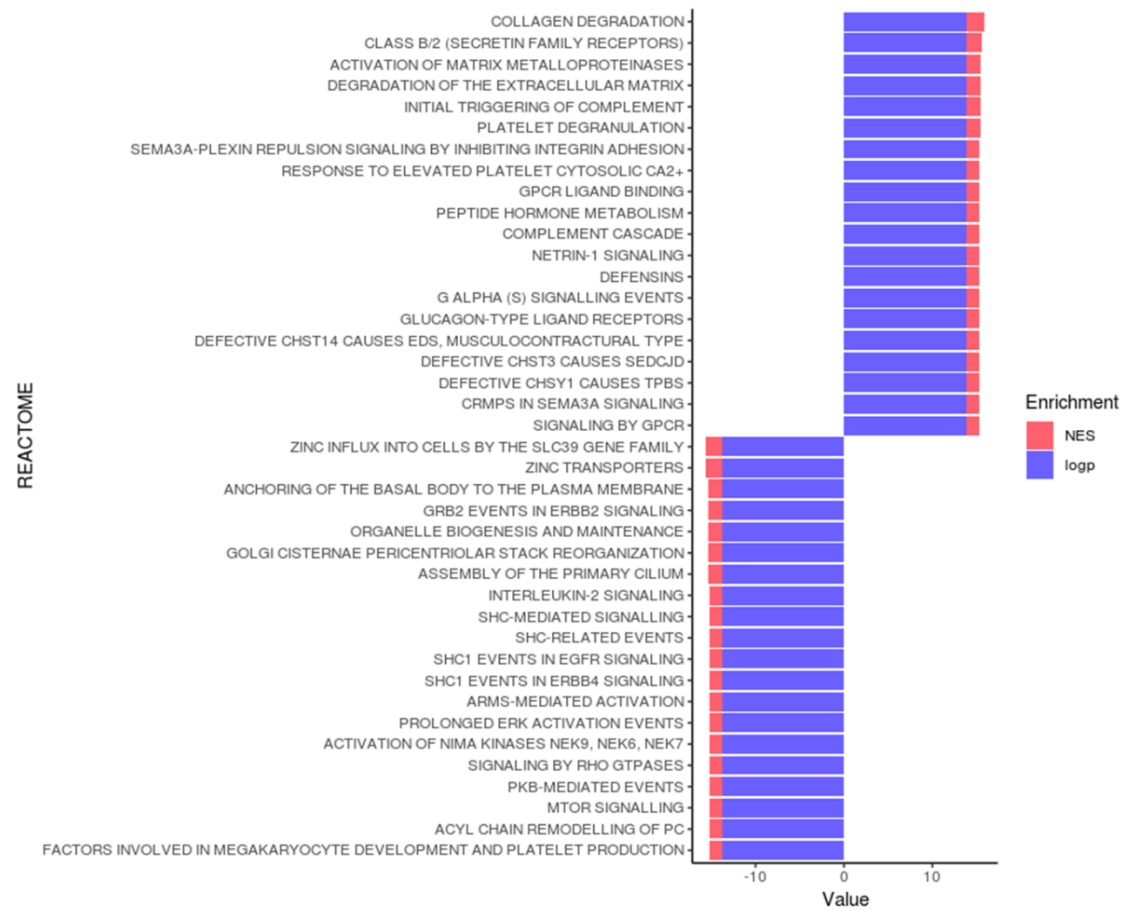

Supplement: S2 Fig — NES is the normalized enrichment score, which is shown as red bars, and the logP is displayed as either the logP or–logP to indicate enrichment or lack of enrichment in the PH group. Positive values indicate enrichment in the PH group. Negative values indicate a lack of enrichment in the PH group, or enrichment in the CO group. (PDF) [file pone.0220573.s005.pdf]

Supplemental Figure 3

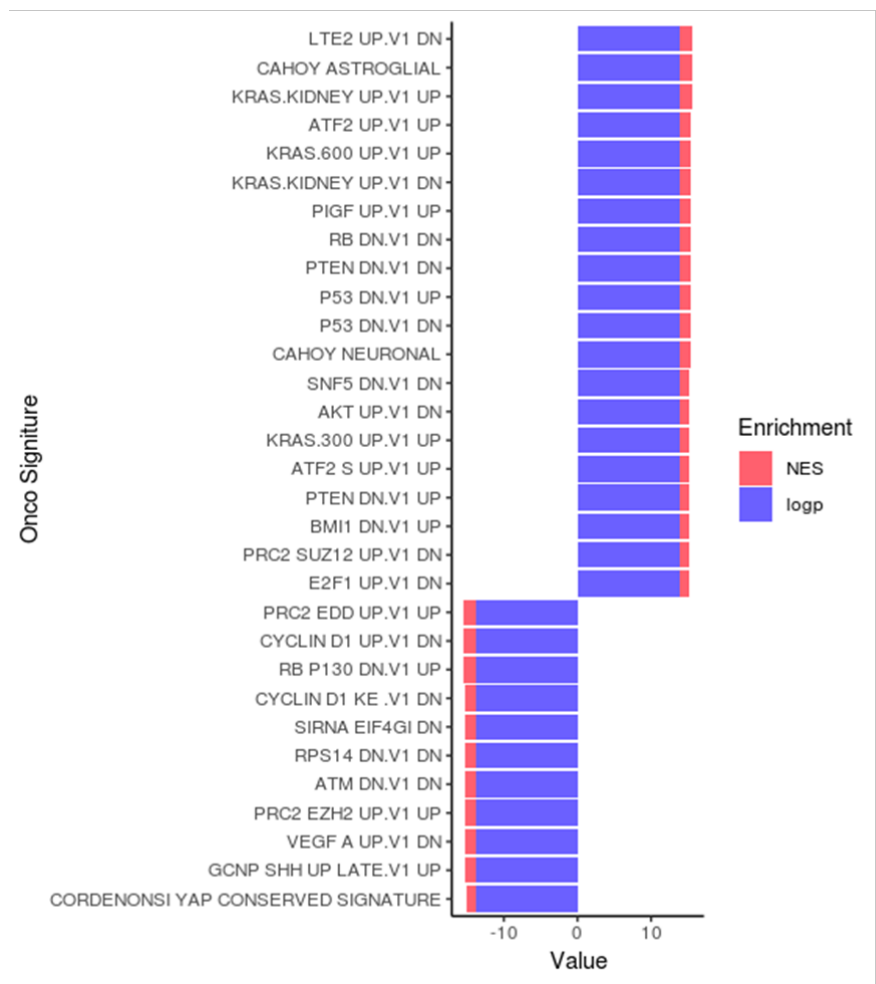

Supplement: S3 Fig — NES is the normalized enrichment score, which is shown as red bars, and the logP is displayed as either the logP or–logP to indicate enrichment or lack of enrichment in the PH group. Positive values indicate enrichment in the PH group. Negative values indicate a lack of enrichment in the PH group, or enrichment in the CO group. (PDF) [file pone.0220573.s006.pdf]

Supplemental Figure 4

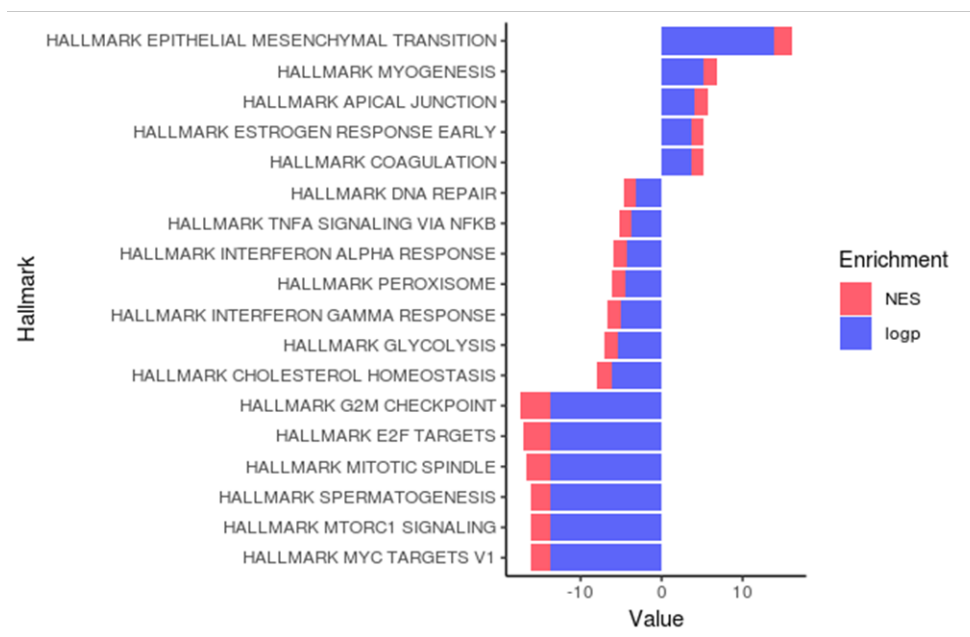

Supplement: S4 Fig — NES is the normalized enrichment score, which is shown as red bars, and the logP is displayed as either the logP or–logP to indicate enrichment or lack of enrichment in the PH group. Positive values indicate enrichment in the PH group. Negative values indicate a lack of enrichment in the PH group, or enrichment in the CO group. (PDF) [file pone.0220573.s007.pdf]

Supplemental Figure 5

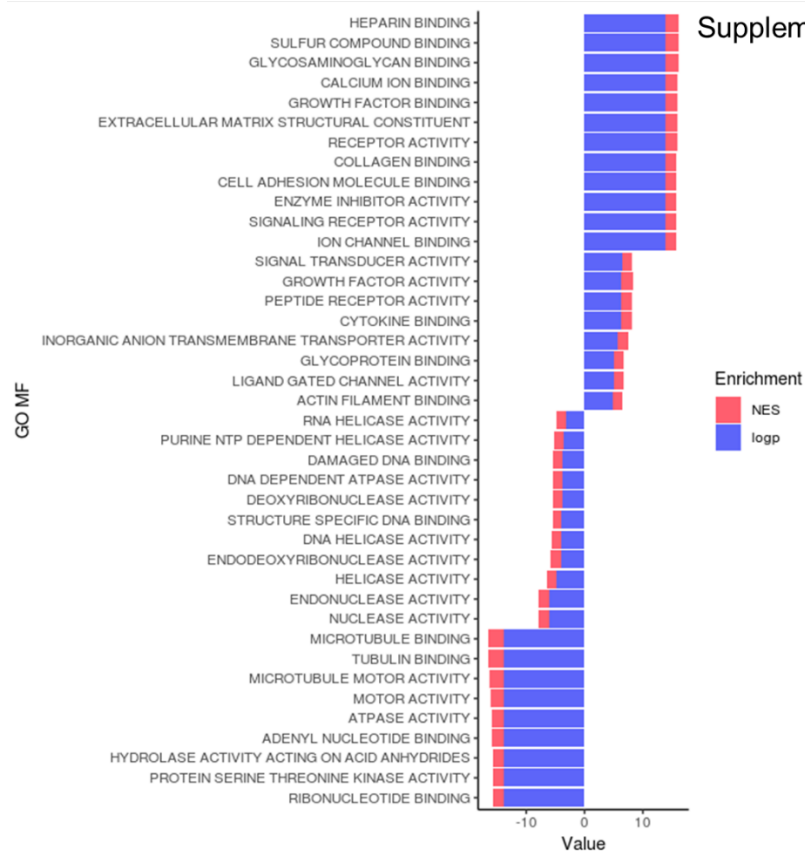

Supplement: S5 Fig — NES is the normalized enrichment score, which is shown as red bars, and the logP is displayed as either the logP or–logP to indicate enrichment or lack of enrichment in the PH group. Positive values indicate enrichment in the PH group. Negative values indicate a lack of enrichment in the PH group, or enrichment in the CO group. (PDF) [file pone.0220573.s008.pdf]

Supplemental Figure 6

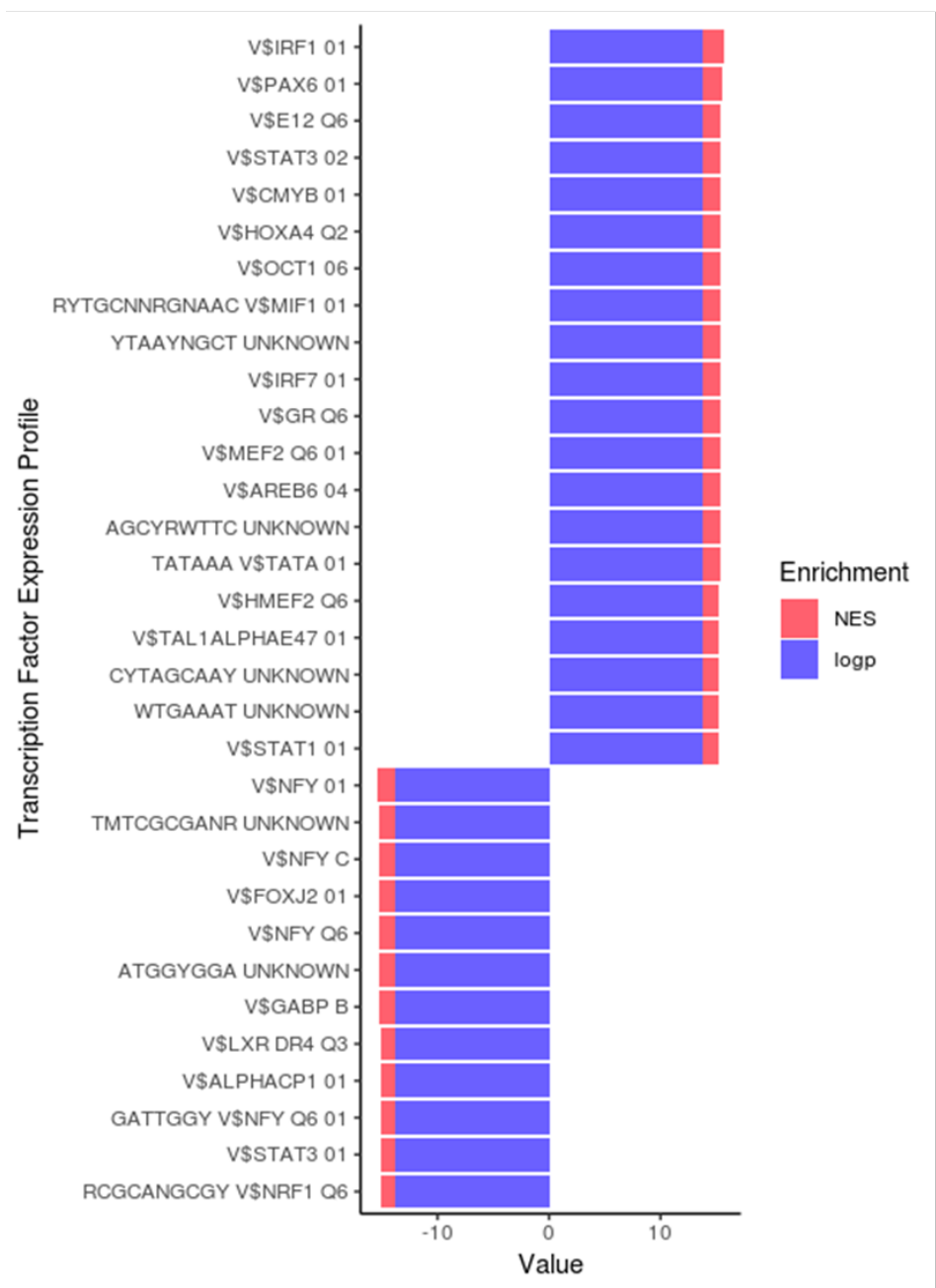

Supplement: S6 Fig — NES is the normalized enrichment score, which is shown as red bars, and the logP is displayed as either the logP or–logP to indicate enrichment or lack of enrichment in the PH group. Positive values indicate enrichment in the PH group. Negative values indicate a lack of enrichment in the PH group, or enrichment in the CO group. (PDF) [file pone.0220573.s009.pdf]

Supplemental Figure 7

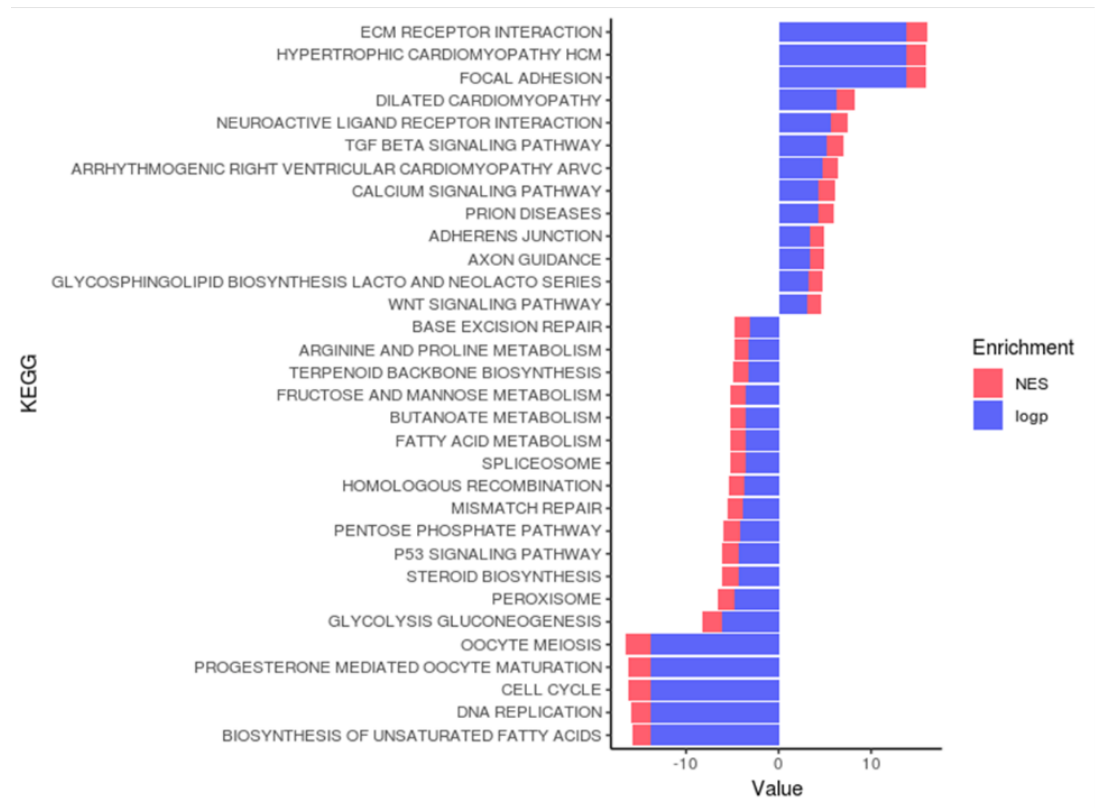

Supplement: S7 Fig — NES is the normalized enrichment score, which is shown as red bars, and the logP is displayed as either the logP or–logP to indicate enrichment or lack of enrichment in the PH group. Positive values indicate enrichment in the PH group. Negative values indicate a lack of enrichment in the PH group, or enrichment in the CO group. (PDF) [file pone.0220573.s010.pdf]

Supplemental Figure 8

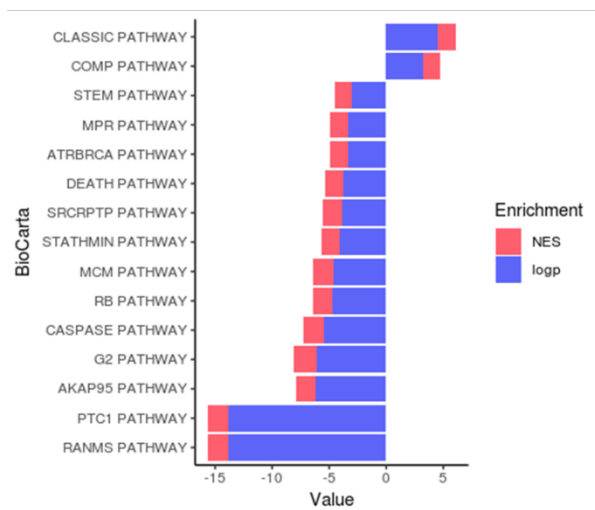

Supplement: S8 Fig — NES is the normalized enrichment score, which is shown as red bars, and the logP is displayed as either the logP or–logP to indicate enrichment or lack of enrichment in the PH group. Positive values indicate enrichment in the PH group. Negative values indicate a lack of enrichment in the PH group, or enrichment in the CO group. (PDF) [file pone.0220573.s011.pdf]

Supplemental Figure 9

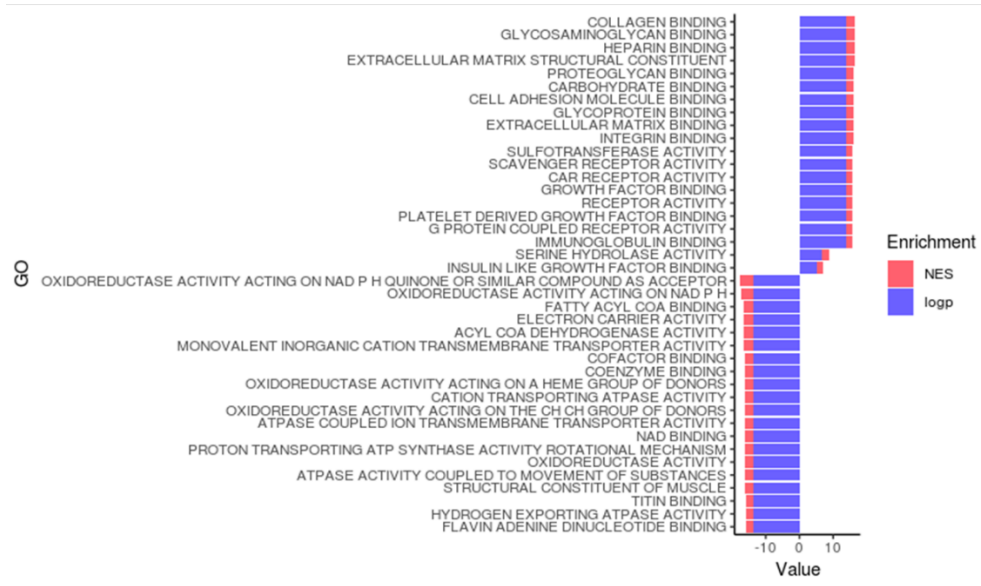

Supplement: S9 Fig — NES is the normalized enrichment score, which is shown as red bars, and the logP is displayed as either the logP or–logP to indicate enrichment or lack of enrichment in the PH group. Positive values indicate enrichment in the PH group. Negative values indicate a lack of enrichment in the PH group, or enrichment in the CO group. (PDF) [file pone.0220573.s012.pdf]

Supplemental Figure 10

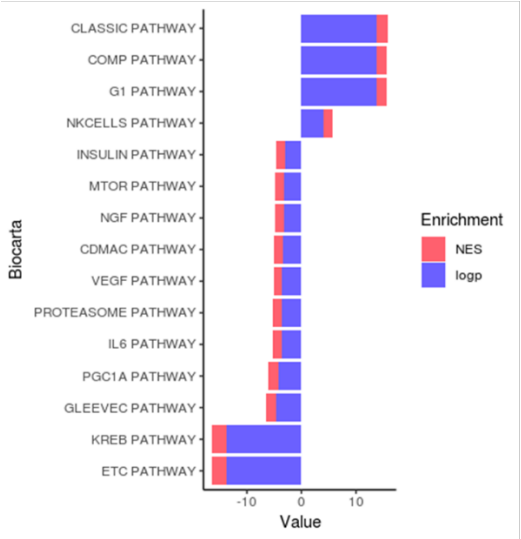

Supplement: S10 Fig — NES is the normalized enrichment score, which is shown as red bars, and the logP is displayed as either the logP or–logP to indicate enrichment or lack of enrichment in the PH group. Positive values indicate enrichment in the PH group. Negative values indicate a lack of enrichment in the PH group, or enrichment in the CO group. (PDF) [file pone.0220573.s013.pdf]

Supplemental Figure 11

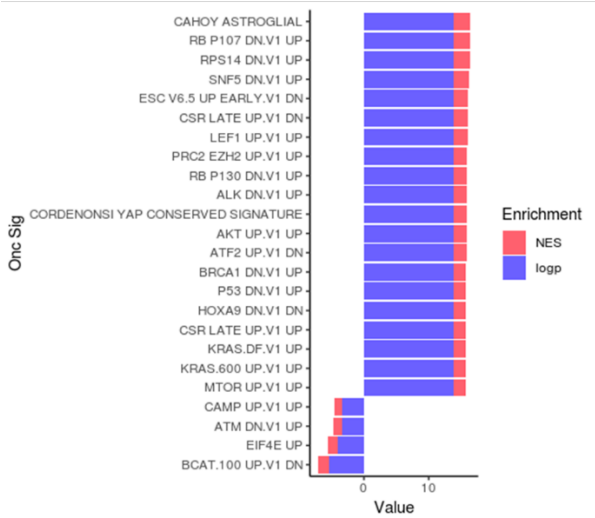

Supplement: S11 Fig — NES is the normalized enrichment score, which is shown as red bars, and the logP is displayed as either the logP or–logP to indicate enrichment or lack of enrichment in the PH group. Positive values indicate enrichment in the PH group. Negative values indicate a lack of enrichment in the PH group, or enrichment in the CO group. (PDF) [file pone.0220573.s014.pdf]

Supplemental Figure 12

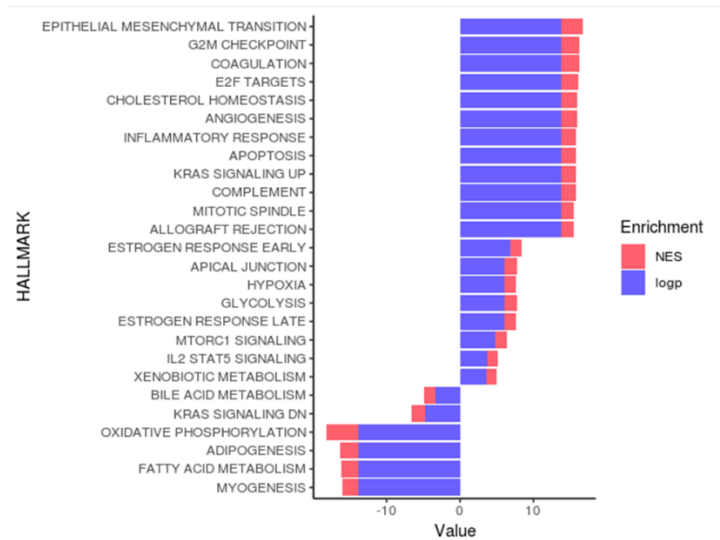

Supplement: S12 Fig — NES is the normalized enrichment score, which is shown as red bars, and the logP is displayed as either the logP or–logP to indicate enrichment or lack of enrichment in the PH group. Positive values indicate enrichment in the PH group. Negative values indicate a lack of enrichment in the PH group, or enrichment in the CO group. (PDF) [file pone.0220573.s015.pdf]

Supplemental Figure 13

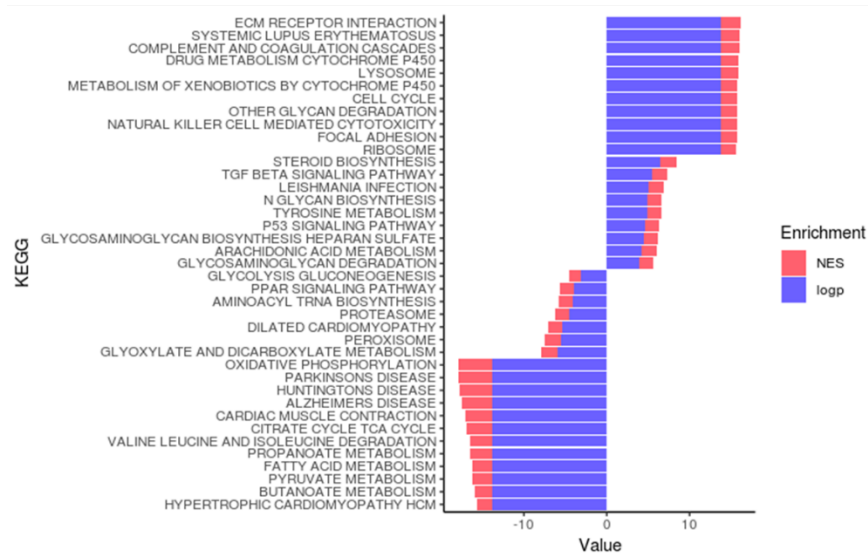

Supplement: S13 Fig — NES is the normalized enrichment score, which is shown as red bars, and the logP is displayed as either the logP or–logP to indicate enrichment or lack of enrichment in the PH group. Positive values indicate enrichment in the PH group. Negative values indicate a lack of enrichment in the PH group, or enrichment in the CO group. (PDF) [file pone.0220573.s016.pdf]

Supplemental Figure 14

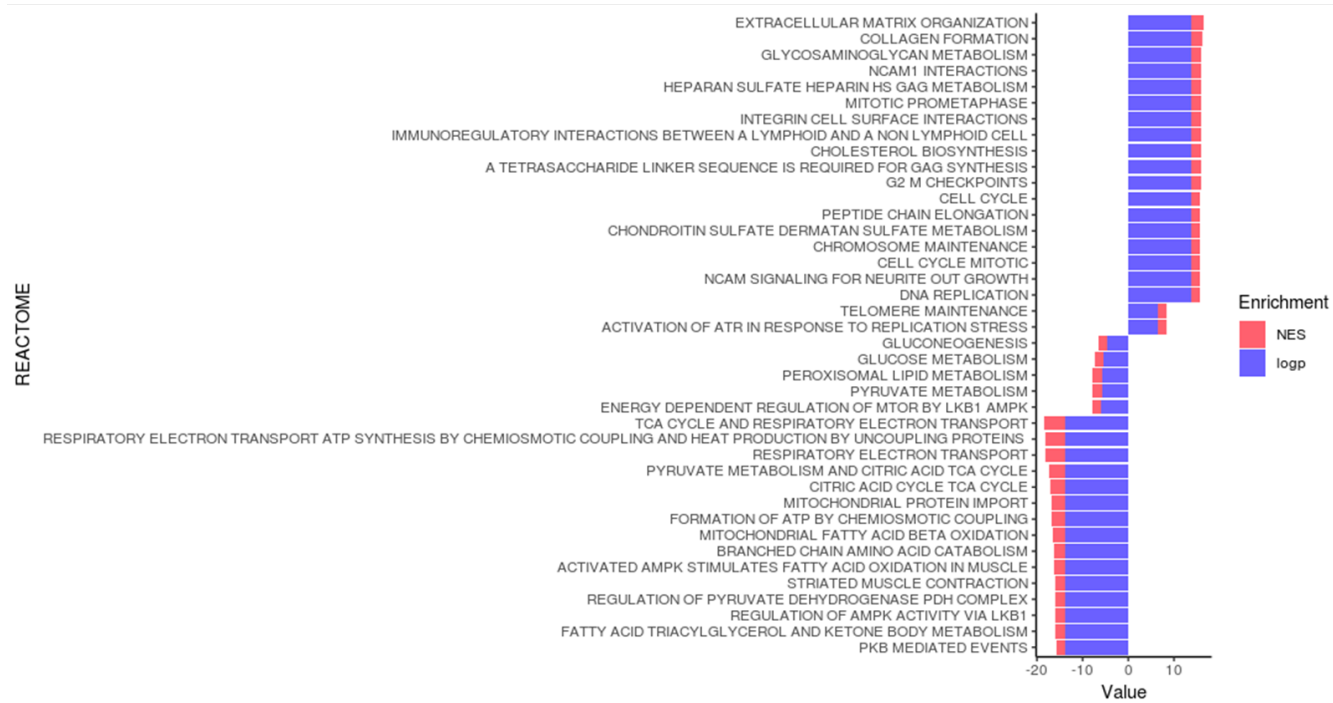

Supplement: S14 Fig — NES is the normalized enrichment score, which is shown as red bars, and the logP is displayed as either the logP or–logP to indicate enrichment or lack of enrichment in the PH group. Positive values indicate enrichment in the PH group. Negative values indicate a lack of enrichment in the PH group, or enrichment in the CO group. (PDF) [file pone.0220573.s017.pdf]
